# Distinction between serological responses following tick-borne encephalitis virus (TBEV) infection vs vaccination, Sweden 2017

**DOI:** 10.2807/1560-7917.ES.2018.23.3.17-00838

**Published:** 2018-01-18

**Authors:** Bo Albinsson, Sirkka Vene, Lars Rombo, Jonas Blomberg, Åke Lundkvist, Bengt Rönnberg

**Affiliations:** 1 Department of Medical Biochemistry and Microbiology, Zoonosis Science Center, Uppsala University, Uppsala, Sweden; 2 Laboratory of Clinical Microbiology, Uppsala University Hospital, Uppsala, Sweden; 3 The Public Health Agency of Sweden, Solna, Stockholm, Sweden; 4 Department of Infectious diseases, Eskilstuna, Sweden; 5 Clinical Research Centre, Sormland county council, Uppsala University, Uppsala, Sweden; 6 Department of Medical Sciences, Uppsala University, Uppsala, Sweden

**Keywords:** Tick-borne encephalitis (TBE), vaccine-preventable diseases, vaccines and immunisation, zoonotic infections, viral encephalitis, diagnostics

## Abstract

Tick-borne encephalitis virus (TBEV) is an important European vaccine-preventable pathogen. Discrimination of vaccine-induced antibodies from those elicited by infection is important. We studied anti-TBEV IgM/IgG responses, including avidity and neutralisation, by multiplex serology in 50 TBEV patients and 50 TBEV vaccinees. Infection induced antibodies reactive to both whole virus (WV) and non-structural protein 1 (NS1) in 48 clinical cases, whereas 47 TBEV vaccinees had WV, but not NS1 antibodies, enabling efficient discrimination of infection/vaccination.

Sweden reported record-high numbers of tick-borne encephalitis (TBE), 391 cases, during 2017. TBE diagnosis is mainly performed by serology. Improved serology should distinguish TBE virus (TBEV) antibodies induced by infection from those induced by vaccination; it should control for cross-reactions and detect suboptimal vaccinations. The major surrogate indicator of protection, neutralising antibodies measured by neutralisation test (NT), requires a biosafety level 3 facility and is time-consuming. We aimed to address all these issues. In this study, we made use of the fact that TBEV NS1 antigen is not present in existing vaccine preparations; thus vaccinees are not expected to develop a serological response against NS1, so that anti-TBE antibodies induced by infection can be distinguished from those induced by vaccination.

## Proof of concept study of immune responses after infection or vaccination

### Serum samples 

We analysed 50 serum samples drawn between 2011 and 2014 from patients in the region of Uppsala Akademiska hospital, Sweden, with clinical suspicion of acute TBEV infection. All had a serological profile consistent with current or recent TBEV infection, i.e. high levels of TBEV-reactive IgM and low or borderline levels of TBEV-reactive IgG in a commercial assay (Siemens Healthcare Diagnostics AG, Marburg, Germany), and were confirmed as TBEV IgM-positive by another commercial test (Reagena OY, Toivala, Finland) (data not shown). 

We also analysed 150 serum samples from 50 healthy individuals who were vaccinated in Eskilstuna, Sweden, between 2012 and 2013 with TBEV vaccine (FSME-immun, Pfizer, New York, United States (US)). Three serum samples per vaccinee were drawn: on day 0, the day of the first dose (n = 50), on day 120 after the first vaccination dose, i.e. a minimum of 30 days after at least two doses (n = 50), and on day 390 after the first vaccine dose, 30 days after at least three doses (n = 50). For all time points after the first dose, a difference of +/− 2 days was accepted.

### Suspension multiplex immunoassay reactivity in acute-phase TBE patients vs TBE vaccinees

TBEV whole virus (WV) antigen was purchased from Jena Bioscience, Jena, Germany (Cat. No. PR-BA112) and TBEV non-structural protein 1 (NS1) antigen from Native Antigen Company, Heyford Park, United Kingdom (Cat. No. TBEV-NS1–100). The TBEV-specific suspension multiplex immunoassay (SMIA) was performed as earlier described for the more comprehensive Flavivirus suspension multiplex immunoassay (FSMIA) [1]. Briefly, each antigen was coupled to carboxylated differentially colour-marked magnetic microspheres using carbodiimide. For IgG determination, serum diluted 1:50 was added to 96-well microtitre plates. Vortexed and sonicated microsphere mixture was added to each well, giving a final serum dilution of 1:100. Subsequently, the microspheres were re-suspended and biotinylated protein G was added, incubated for 30 min, washed and re-suspended, followed by the addition of streptavidin–phycoerythrin (SA-PE) at a concentration of 4 μg/mL, and finally incubated for 15 min. The microspheres were washed once before re-suspension and analysis in a Luminex 200 instrument. For IgM determination, serum was pre-incubated with GullSORB (Meridian Life Science, Memphis, US) to remove IgG. Each well was subsequently incubated with microsphere mixture, followed by addition of biotinylated anti-IgM and SA-PE conjugate as described above for detection of IgG. The avidity index (AI) was calculated as the ratio between the median fluorescence intensity (MFI) after and before treatment with 8 M urea.

The assay cut-offs (IgM/WV = 250; IgM/NS1 = 200; IgG/WV = 250, IgG/NS1 = 200) for each antigen and antibody isotype combination were calculated as the average MFI plus at least 3 standard deviations (SD) of 70 Swedish TBEV antibody-negative sera.

#### Suspension multiplex immunoassay IgM reactivity 

**Figure 1:**
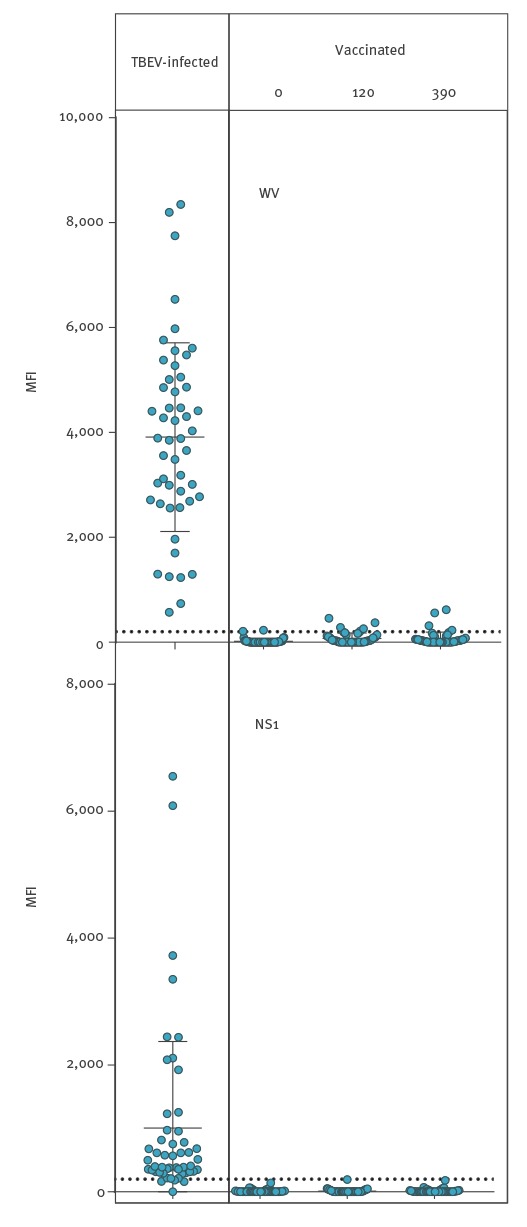
Suspension multiplex immunoassay for IgM reactivity with tick-borne encephalitis whole virus vs non-structural protein 1 antigens in infected (n = 50) vs vaccinated individuals (n = 50) at 0, 120 and 390 days after first vaccination, Sweden, 2017

All 50 acute-phase TBE samples showed WV-specific IgM. Forty-six of 50 patients also had detectable levels of NS1-specific IgM (Figure 1, Table). Seven serum samples from vaccinees had a weak IgM response to WV. Only one of the 150 samples from vaccinees gave an IgM reaction to NS1 slightly over the cut-off value of 200 (202 MFI).

MFI: median fluorescence intensity; NS1: non-structural protein 1; TBEV: tick-borne encephalitis virus; WV: whole virus.

Upper panels: whole virus; lower panels: NS1. Cut-offs are shown by dotted lines. Averages and standard deviations are also plotted.

**Table:** Tick-borne encephalitis virus reactivity of sera from acutely infected patients (n = 50) vs vaccines (n = 50), at 0, 120 and 390 days after first vaccination, Sweden, 2017

Category	anti-WV IgM^a^	anti-NS1 IgM^b^	anti-WV IgG^a^	anti-NS1 IgG^b^
**Infected **				
n = 50**^c^**	50^d^	46^e^	50	43^f^
**Vaccinated **				
Day 0 (n = 50)	0	0	2	0
Day 120 (n = 50)	4	1	37	1
Day 390 (n = 50)	3	0	47	2

MFI: median fluorescence intensity; NS1: non-structural protein 1; TBEV: tick-borne encephalitis virus; WV: whole virus.

**^a^** Positive: ≥ 250 MFI.

**^b^** Positive: ≥ 200 MFI.

^c^ A total of 48 of the 50 sera from patients with acute TBEV infection had IgM and/or IgG antibody reactivity to NS1.

**^d^** All 50 samples from patients with acute TBEV infection had detectable levels of IgM to WV. The maximum number of positive IgM reactions to WV after vaccination at one of the three time points was four (at 120 days). Three vaccinees were IgM-positive at 390 days (two of them were also positive at 120 days). The difference between 50 of 50 infected and four of 50 vaccinated was significant (p < 0.0001, Fisher’s exact test, two-tailed).

**^e^** The number of positive IgM reactions to NS1 during acute TBEV infection was 46. One of the samples from a vaccinee was positive. The difference between 46 of 50 infected and one of 50 vaccinated was significant (p < 0.0001, Fisher’s exact test, two-tailed).

**^f^** The difference between 43 of 50 infected and two of 50 vaccinated positive for IgG to NS1 at day 390 was significant (p < 0.0001, Fisher’s exact test, two-tailed).

#### Suspension multiplex immunoassay IgG reactivity 

**Figure 2:**
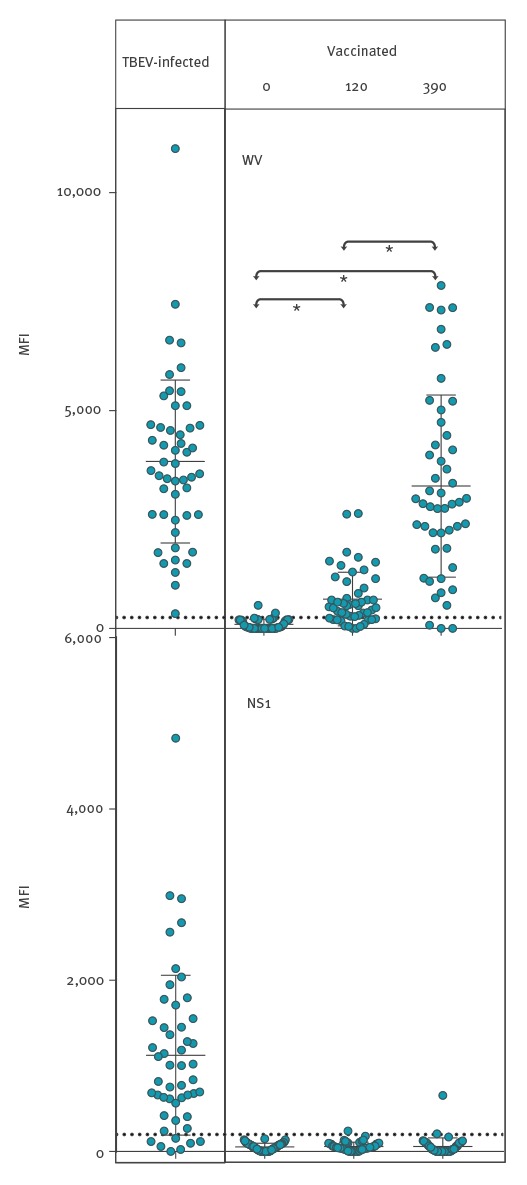
Suspension multiplex immunoassay for IgG reactivity with tick-borne encephalitis whole virus vs non-structural protein 1 antigens in infected (n = 50) vs vaccinated individuals (n = 50) at 0, 120 and 390 days after first vaccination, Sweden, 2017

All 50 samples from acute-phase TBE patients had WV-specific IgG and 43 also had detectable levels of NS1-specific IgG (Table, Figure 2). After the booster immunisations, the IgG reactivities to WV increased on average 14-fold among 47 of the 50 vaccinees (Table, Figure 2). In contrast, the IgG reactivity to NS1 was negative in 147 of the 150 serum samples from vaccinees. Vaccination induced an IgG response to WV of more than 250 MFI in 47 of 50, and of more than 1,000 in 43 of 50 samples at day 390. Three vaccinees did not respond to the vaccine according to SMIA. 

MFI: median fluorescence intensity; NS1: non-structural protein 1; TBEV: tick-borne encephalitis virus; WV: whole virus.

Upper panels: whole virus; lower panels: NS1. Cut-offs are shown by dotted lines. Averages and standard deviations are also plotted.

* Significant differences between groups, exceeding three standard deviations (Mann–Whitney test, p < 0.0001).

### Comparison of antibody responses following infection vs vaccination

All 50 samples from patients with acute TBEV infection had detectable levels of IgM to WV. The maximum number of positive IgM reactions to WV after vaccination at one of the three time points was four (at 120 days). Three vaccinees were IgM-positive at 390 days (two of them were also positive at 120 days). The difference between 50 of 50 infected and four of 50 vaccinated was significant (p < 0.0001, Fisher’s exact test, two-tailed) (Table). The number of positive IgM reactions to NS1 during acute TBEV infection was 46 of 50. Only one of the 150 samples from 50 vaccinees was positive. The difference between 46 of 50 infected and one of 50 vaccinated was significant (p < 0.0001, Fisher’s exact test, two-tailed) (Table). The difference between 43 of 50 infected and two of 50 vaccinated positive for IgG to NS1 at day 390 was significant (p < 0.0001, Fisher’s exact test, two-tailed) (Table).

### Neutralising antibodies and avidity index

**Figure 3:**
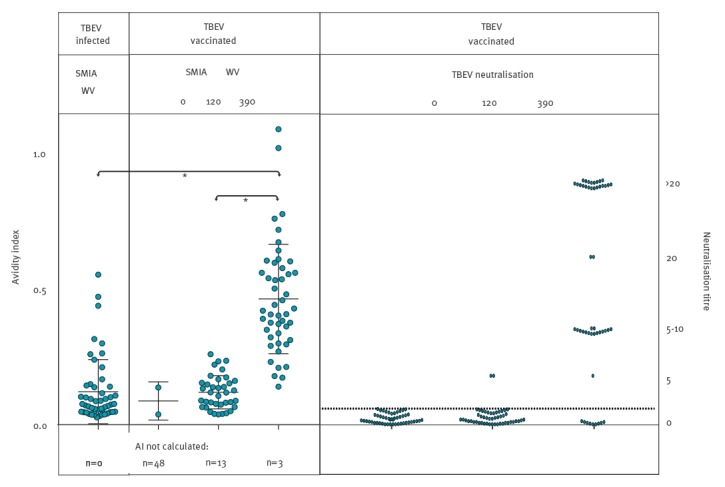
Suspension multiplex immunoassay avidity index for IgG against tick-borne encephalitis whole virus in infected patients (n = 50) vs vaccinees (n = 50), and neutralisation titres at 0, 120 and 390 days after the first vaccination, Sweden, 2017

TBEV neutralisation titres were determined essentially as previously described [2,3]. Briefly, serum samples, including positive and negative controls, diluted 1:5, were further diluted serially in 96-well tissue culture plates and infected with ca 50 FFD_50_ (50% focus-forming doses) of TBEV. After incubation, ca 5 x 10^5^ BHK-21 S13-cells/mL were added to each well. Virus foci were visualised by an anti-TBEV monoclonal antibody, followed by a fluorescent secondary antibody conjugate. Neutralising antibody titres were calculated as the reciprocal of the serum dilution that reduced the challenge virus to one FFD_50_. All 50 samples from vaccinees drawn at day 0 were found negative, two of the 50 samples drawn at day 120 were positive (titre 5), while 43 of 50 samples drawn at day 390 were positive (titres 5 to >20) (Figure 3, right panel).

MFI: median fluorescence intensity; NS1: non-structural protein 1; TBEV: tick-borne encephalitis virus; WV: whole virus.

The cut-off value is shown by a dotted line. Averages and standard deviations are also plotted.

* Group differences which were significant, exceeding three standard deviations (Mann–Whitney test, p < 0.0001).

The IgG AI against WV was low (mean: 0.12; SD = 0.12) for most of the 50 patients with acute TBEV infection, as expected early in the course of the infection (Figure 3, left panel). Figure 3 (middle panel) shows a pronounced increase in AIs among the vaccinees. At the 390 day time point (mean: 0.46; SD = 0.20), their AI values were higher than in the acute-phase TBE patients.The difference was significant (Mann–Whitney test of groups, p < 0.0001). Figure 3 illustrates that the higher avidity, as well as the higher IgG reactivity to WV (Figure 2), corresponded well to the higher NT titres at day 390. Among all 150 samples from vaccinees, 34 of 43 NT-positive samples had an AI exceeding 0.3, whereas only three of 107 NT-negative samples had an AI exceeding 0.3 (Fisher’s exact test, p < 0.0001). 

## Discussion

TBEV is a member of the Flavivirus genus of the *Flaviviridae* family [4]. Other well-known and important human pathogens among the flaviviruses include dengue, Japanese encephalitis, yellow fever, and Zika viruses. TBE is prevalent in large areas of Europe and in parts of Asia [5]. The clinical disease is typically bi-phasic, with an initial influenza-like period, in 25% followed by a second phase of meningoencephalitis/encephalitis affecting the central nervous system (CNS) [4]. TBEV gives a serious infection with a fatality rate of 1–2% in Europe, with neurological sequelae in ca 20% of those with CNS symptoms. As a response to the rising number of TBEV infections [6], an increasing number of TBE vaccinations are administered in Sweden as well as in many other European countries [5,7].

Owing to the very low amount, or in most cases absence, of detectable TBEV RNA at the onset of the CNS symptoms in immunocompetent patients, serology is required for TBEV diagnostics [4]. Serological discrimination between vaccine-induced antibodies and those elicited by acute infection, and measuring of immune responses after vaccination are important. Although our results indicated that the TBEV SMIA is more sensitive than the commercial assay (ELISA) for detection of WV-specific IgG, larger serum panels are needed for a detailed evaluation. Moreover, cross-reactions between members of the genus Flavivirus must be compensated for using the more comprehensive FSMIA [1]. 

The highly immunogenic NS1 is not present in the inactivated whole virus preparations of the TBEV vaccines available in the European Union and European Economic Area (EU/EEA) (FSME-immun (Pfizer) and Encepur (Glaxo Smith Kline)). This offered a possibility to efficiently distinguish antibodies induced by vaccination from those induced by infections.

There has been some controversy regarding the number of TBEV vaccine doses needed to achieve protection, and at which intervals booster doses should be given. Based on a Swedish study of the serological response in 535 persons after TBE vaccination [8], the Swedish guidelines from 2008 raised the interval for booster injections from 3 to 5 years after four initial doses. The IgG reactivities to NS1 in three samples from vaccinees in our study are not fully understood. One possible explanation is that these vaccinees may have had an abortive TBEV infection during the vaccination period.

The protection rate of the FSME-immun vaccine has been estimated at 96–99% according to field studies in Austria [9]. A study from 2010 presented data from 27 Swedish patients with clinical symptoms and signs of TBE, together with serological evidence of TBEV infection despite vaccination [10]. These vaccination failures, characterised by a slow and initially non-detectable development of TBEV-specific IgM, despite a rapid rise of IgG and neutralising antibodies in serum, might be more common than known to date. In our present study, three vaccinees did not develop detectable levels of TBEV-specific IgG or neutralising antibodies. Our SMIA is therefore likely to be valuable also for rapid and efficient detection of vaccination failures, which will now be further investigated.

## Conclusion

The best surrogate markers for protection are TBEV neutralising antibodies, measured by NT [7,9]. The high proportion of AI values above 0.3 among the neutralising sera suggested that AI could be used to predict protection against TBEV, offering an alternative to the handling of infectious virus inherent to NT, which requires biosafety level 3 facilities. In line with the NT results, we have shown here that at least three immunisations are necessary to achieve a high avidity as measured by the SMIA. Our new method will be an effective tool in clinical diagnostics including vaccine failure investigations and in studies of seroprevalence/population immunity. Furthermore, combined with measurement of avidity, the method has a potential to provide a surrogate marker for protection against TBEV infection.
